# What Is Your Diagnosis? Dermal Mass From the Left Forelimb of a Dog

**DOI:** 10.1111/vcp.70057

**Published:** 2025-10-07

**Authors:** Heather D. McDavid, Miranda D. Vieson, Robert Armentano, Nicholas T. Noto

**Affiliations:** ^1^ University of Illinois College of Veterinary Medicine Urbana Illinois USA; ^2^ Veterinary Diagnostic Laboratory University of Illinois College of Veterinary Medicine Urbana Illinois USA; ^3^ Veterinary Specialty Center Bannockburn Illinois USA

**Keywords:** canine, dermatopathology, follicular hamartoma

## 1 Case Presentation

An 8‐year‐old male neutered Dalmatian presented to a specialty referral hospital for a recheck of ammonium urate uroliths secondary to hyperuricosuria. The patient's urinary stones have been well managed with low‐dose allopurinol and a urinary diet since April of 2023. At the current recheck, the owners noted a dermal mass on the left forelimb that had been recently growing. The clinician described a 1.4 × 1.4 cm dermal mass on the dorsal left carpus with no associated lameness or pain. Additional physical examination abnormalities included a small alopecic area on the dorsum of the head and a mildly bowlegged hind stance. The patient has a history of a hemangioma removed from the dermis just cranial to the prepuce in November of 2022.

A fine‐needle aspirate of the left forelimb mass was submitted for cytologic evaluation (Figure 1). The clinician noted that the mass was very firm, and it was difficult to retrieve material from the mass upon aspiration.

**FIGURE 1:**
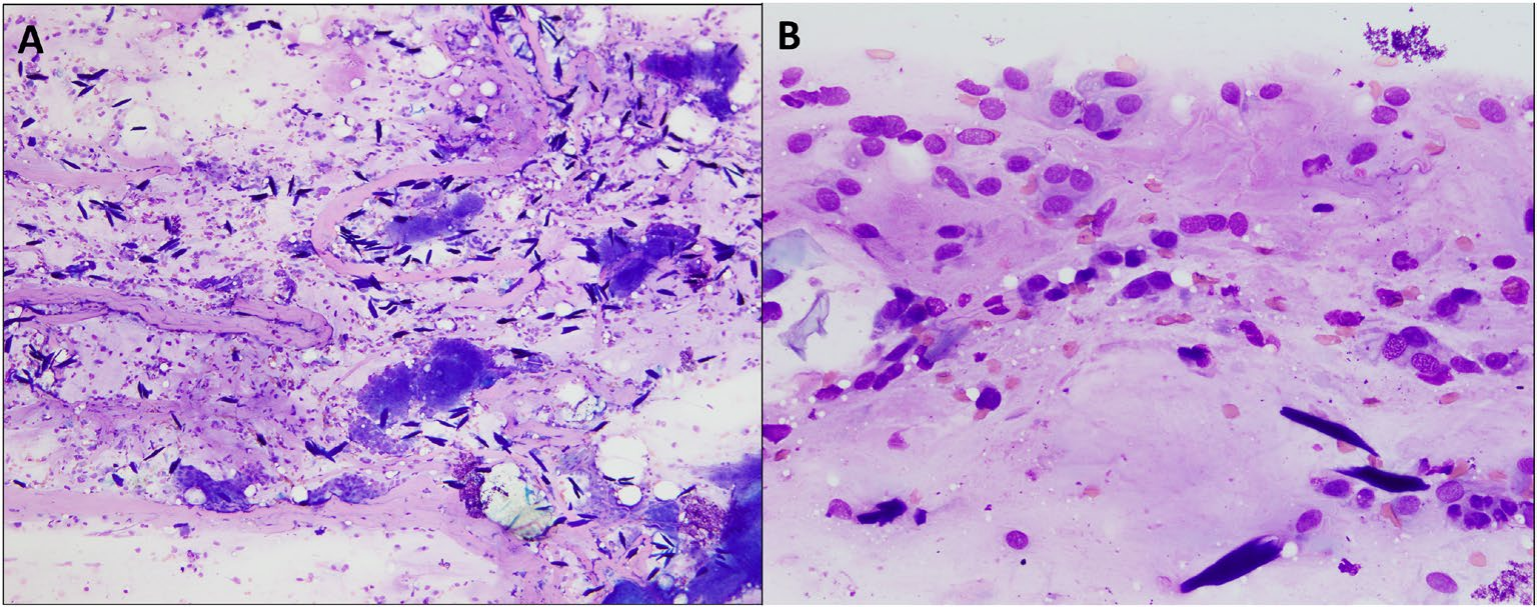
Representative cytologic appearance of a dermal mass on the left forelimb of an 8‐year‐old Dalmatian. Wright‐Giemsa stain. (A) ×10 objective, (B) ×50 objective.

### 1.1 Cytologic Interpretation: Atypical Mesenchymal Proliferation With Possible Epithelial Proliferation

On cytologic evaluation, the sample was of moderate intact cellularity and contained a minimal amount of peripheral blood. A population of mildly to moderately atypical mesenchymal cells was arranged primarily individually. They were spindloid to stellate in shape with a moderate amount of basophilic cytoplasm, round to ovoid nuclei with stippled chromatin, and one to few small but prominent nucleoli. These cells displayed mild to moderate anisocytosis and anisokaryosis, common binucleation, and rare nuclear molding. An additional population of densely grouped and variably preserved cells was identified. While many features of these cells were obscured, multiple small and prominent nucleoli were noted. Although these cells appeared to be arranged in dense clusters, they appeared spindloid at the edge of the clusters, and both epithelial and mesenchymal origins were considered. The background contained a striking amount of fibrillar collagen, a moderate amount of mature keratin, and occasional bare nuclei from ruptured cells.

Cytologic findings were interpreted as atypical mesenchymal proliferation with possible epithelial proliferation. Differential diagnoses for the atypical mesenchymal cells included a neoplastic population (likely benign) and reactive fibroblasts. However, given the large amount of collagen, mature keratin, and possible epithelial groups, a sebaceous or fibroadnexal hamartoma was also offered as a differential diagnosis.

## 2 Additional Results

Biopsy with histopathology of the left forelimb mass was elected. On histopathologic evaluation, there was a dome‐shaped appearance to the epidermis due to distention of multiple follicular infundibula by clear space and widening of follicular ostia (Figure 2). The hair follicles were slightly thickened and elongated with enlarged hair bulbs and thickened shafts (Figure 3). Rarely, the follicles were surrounded by a few concentric layers of fibrosis. The microscopic diagnosis was follicular hamartoma.

**FIGURE 2:**
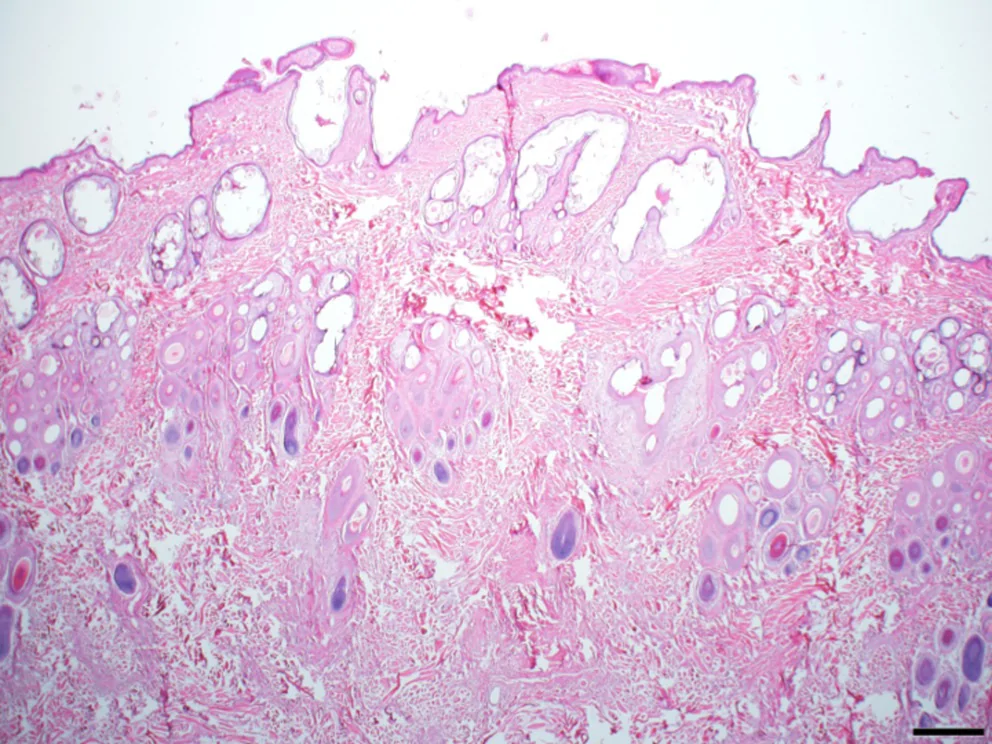
There is a subtle dome‐shaped profile to the epidermis attributed to distension of the multiple follicular infundibula by clear space and widening of follicular ostia, creating a digitated appearance to the surface. H & E stain, Bar = 500 μm.

**FIGURE 3:**
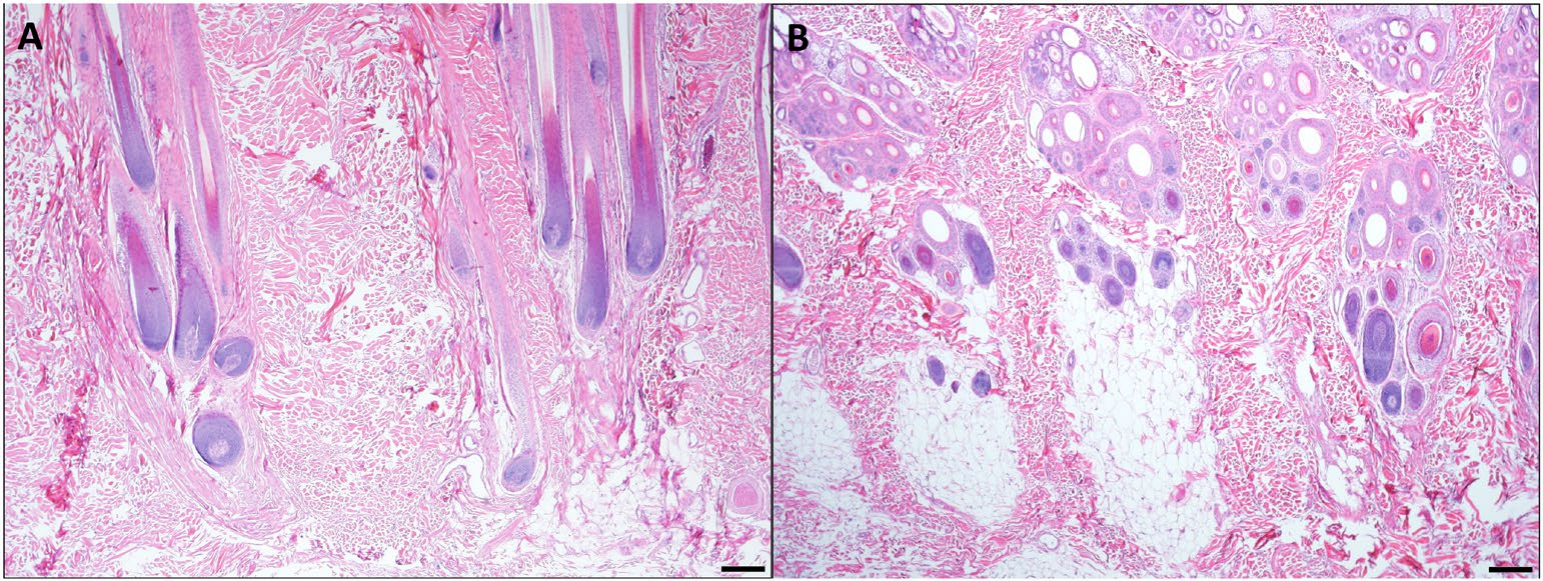
Hair follicles within the mass effect (A) tended to be thickened, elongated, and enlarged when compared to clusters of hair follicles at the edge of the mass effect (B). H & E stain, Bar = 200 μm.

## 3 Discussion

Follicular hamartomas (FH), also known as follicular nevi, are non‐neoplastic [1] and have been referred to as “cosmetic blemishes”[2]. FHs have no known etiology, and breed, sex, or site predilection has not been described [1]. They are very uncommon in animals [1, 2]; in a study documenting 81 cases of nevi and cutaneous hamartomas in dogs, only two dogs were diagnosed with FHs, while the others had more common lesions such as fibroadnexal hamartomas [3]. If left untreated, FHs may predispose the animal to secondary infections and can become regionally extensive; therefore, surgical excision [1, 2, 3] may be elected. The gold standard for diagnosis is surgical biopsy with histopathology [1].

Grossly, these lesions are typically thick, irregular, plaque‐like nodules that can be several centimeters in diameter [1, 2]. Over time, these nodules can increase and expand in size. The thickened skin has been compared to having the appearance of an orange peel [1] or even tiny pebbles [2]. Furthermore, the lesions feel firm and have the texture of a brush due to the accumulation of many follicles [1, 2].

Histologically, FHs are composed of clusters of anagen hair follicles that, while normal in architecture, are larger and more extensive than usual. These follicles usually resemble primary hair follicles [1, 2] that all look similar in appearance [1] with thickened hair shafts emerging from dilated follicular infundibula [2, 3]. Excess keratin is often seen throughout [2], and the larger follicles are frequently associated with larger and more numerous sebaceous glands [1, 3]. Extensive fibroplasia [1, 3] and collagen accumulation have also been reported [1, 2, 3].

Cytologic findings of hamartomatous lesions have been described [4], but published cytology images from an FH were not available to the authors' knowledge. While FHs are primarily diagnosed through histopathology [1], certain cytologic features can lead to a differential diagnosis of FH. Cytologic evaluation of this lesion showed large amounts of collagen and keratin, both of which are commonly seen on histopathologic evaluation of FHs. Fibroplasia is also a common histopathologic finding in FHs. The mesenchymal cells in this case displayed mild atypia and were primarily individualized. Initially, these cells raised concern for a possible neoplastic population; however, these cells most likely represented reactive fibroblasts given the histopathologic diagnosis of FH. Atypical mesenchymal cells are frequently seen cytologically in cases of both fibroplasia and mesenchymal neoplasms, which creates a common diagnostic dilemma. Features more suggestive of fibroplasia, rather than mesenchymal neoplasia, include individualized cells, a low degree of atypia, and the presence of inflammation, while features more suggestive of mesenchymal neoplasia include large aggregates of cells, multinucleation, and anisokaryosis [5].

While the characteristic features described are not specific to FHs, recognizing this combination of cytologic features in conjunction with the clinical characteristics of the lesion allows for a quicker and less invasive method of reaching a differential diagnosis of FH. This is important as these lesions carry a great prognosis following complete excision, particularly when compared to mesenchymal neoplasms which often have similar cytologic features.

## Conflicts of Interest

The authors declare no conflicts of interest.
